# Epidemiology and clinical presentation of kidney amyloidosis have changed over the past three decades: a nationwide population-based study

**DOI:** 10.1186/s12882-025-04136-w

**Published:** 2025-06-02

**Authors:** Hilde J. Vasstrand, Melinda Raki, Rannveig Skrunes, Sabine Leh, Janne Thomsen, Helga Gudmundsdottir, Arnljot Tveit, Anders Hartmann, Anna V. Reisæter, Anders Åsberg, Tale N. Wien

**Affiliations:** 1 Department of Internal Medicine and Department of Medical Research, Bærum Hospital, Vestre Viken Hospital Trust, Drammen, Norway; 2 Institute of Clinical Medicine, Faculty of Medicine, University of Oslo, Oslo, Norway; 3 Department of Pathology, Oslo University Hospital, Rikshospitalet, Oslo, Norway; 4 Department of Medicine, Haukeland University Hospital, Bergen, Norway; 5 Department of Clinical Medicine, University of Bergen, Bergen, Norway; 6 Department of Pathology, Haukeland University Hospital, Bergen, Norway; 7 Department of Nephrology, Oslo University Hospital, Ullevål, Oslo Norway; 8 Department of Transplantation Medicine, Section of Nephrology, Oslo University Hospital, Rikshospitalet, Oslo, Norway; 9 The Norwegian Renal Registry, Oslo University Hospital, Rikshospitalet, Oslo, Norway; 10 Department of Pharmacy, University of Oslo, Oslo, Norway

**Keywords:** Amyloidosis, Clinical presentation, Epidemiology, Amyloid typing, Kidney biopsy, Renal amyloidosis

## Abstract

**Background:**

Early diagnosis of kidney amyloidosis is essential for optimal treatment and improved outcomes. This large, nationwide cohort spanning three decades, explores the changing epidemiology and clinical presentation of kidney amyloidosis in Norway, aiming to raise amyloid awareness.

**Methods:**

In the 30-year period (1988–2017), we identified 479 patients with biopsy-confirmed kidney amyloidosis from national registries. Past medical records were reviewed for additional amyloid relevant data and cases were divided into groups of non-AA and AA amyloidosis.

**Results:**

Overall amyloid biopsy incidence in the registries was stable around 4%, but a shift in types occurred. The AL-dominated non-AA group increased from 1.9% to 2.8% (*p* = 0.014) while the AA group decreased from 2.6% to 1.3% (*p* < 0.001). The change in AA was related to less rheumatic disease, partly compensated by an increase in AA in people who inject drugs. The scope and accuracy of amyloid typing improved in the study period, significantly reducing undetermined cases (*p* < 0.001) and providing more robust diagnoses. Clinical presentation was diverse, but proteinuria was present in 94%. Non-AA patients more often than AA had nephrotic syndrome (70% vs 51%, *p* < 0.001) and better-preserved kidney-function (median (IQR) eGFR 53(55) vs 27(34) ml/min/1.73 m^2^, *p* < 0.001). AA patients were younger (*p* < 0.001) with higher prevalence of hypertension (53% vs 38%, *p* < 0.001). Notably, AA in people who inject drugs was more advanced and near half presented with end-stage kidney disease. In recent years, non-AA presented with significantly improved serum albumin (*p* = 0.002), haemoglobin (*p* = 0.020) and erythrocyte sedimentation ratio (*p* = 0.029). Additionally, the percentage of non-AA with end-stage kidney disease fell from 26.8% to 8.7% (*p* = 0.005), possibly indicating earlier diagnosis.

**Conclusion:**

The epidemiology of kidney amyloidosis has changed over the past 30 years. Biopsy incidence of non-AA is increased, and findings may suggest an earlier diagnosis. Amyloid typing has improved over time and is reflected in more precise amyloid diagnoses and reduced number of undetermined cases in recent years. Although AA related to rheumatic disease is declining, AA amyloidosis in people who inject drugs represents a growing challenge. The changing epidemiology of kidney amyloidosis may impact clinical presentation and future healthcare needs, emphasising the need for amyloid awareness.

**Supplementary Information:**

The online version contains supplementary material available at 10.1186/s12882-025-04136-w.

## Introduction

The amyloidoses are progressive and fatal protein deposition diseases [1]. A common denominator of all amyloidoses is the process where proteins normally present in the human body undergo a conformational change and deposit extracellularly as amyloid fibrils [2]. These fibrils displace normal tissue and may have a toxic effect, resulting in gradual organ failure [2, 3].

The type of protein deposited is important, as the disease course, prognosis and treatment depend on the precursor protein [4, 5]. More than 40 different proteins can cause amyloidosis in humans [6]. The predominant amyloid types affecting the kidney are AA amyloidosis (AA) and AL amyloidosis (AL), but 12 further, rare forms of kidney amyloidosis have been described [6]. AA almost invariably affects the kidney [5], while AL affects the kidney in approximately 70% of cases [7]. AA is a complication of chronic inflammation, with amyloid deposits derived from the acute phase reactant serum amyloid A (SAA) [8]. The amyloid deposited in AL is derived from immunoglobulin light chains, and the underlying disease is a clonal plasma cell disorder [9]. Consequently, these diseases constitute distinct clinical entities necessitating entirely different treatment strategies [4].

The clinical picture in kidney amyloidosis can be heterogenic with symptoms from both the kidneys and other organs [2, 5, 10]. Nephrotic syndrome is present in more than 50% at diagnosis [11–13], but the kidney presentation of the disease can also be subtle with varying degrees of proteinuria and insidious reduction of kidney function [5, 14, 15]. Many kidney diseases have the same presentation, and only a tissue biopsy can confirm the diagnosis [5]. Vague, ambiguous, and multi-systemic symptoms and diagnosis relying on a biopsy, may explain why amyloidosis often is diagnosed late [4, 16] and is underdiagnosed [1]. Early diagnosis is essential for optimized treatment and improves long-term outcomes [4], thus disease awareness among all clinicians is important [7, 16, 17].

Earlier studies of amyloidosis have revealed geographical differences [3] and changes over time [4]. In developed countries, there has been a shift from AA to AL as the dominant type [2], while AA is still the most frequent type in developing countries [18]. This shift is mainly due to better treatment of inflammatory diseases, e.g. rheumatoid arthritis, reducing incidence of AA, despite an increase of AA in people who inject drugs (PWID) [19–22]. Improved diagnostic tools have also influenced the apparent incidence of different types [23, 24]. Some studies suggest changes in demography, such as an aging population, may influence disease occurrence [25, 26]. However, larger population-based studies addressing these aspects in kidney amyloidosis are scarce.

Knowledge of the clinical presentation, disease epidemiology, and temporal trends is essential for clinicians when seeking to identify amyloidosis patients earlier. In this nationwide cohort spanning 30 years, we identify clinical and epidemiological features of kidney amyloidosis, explore changes over time and highlight key characteristics to raise awareness about amyloidosis.

## Material and methods

### Study population and data source

**Fig. 1:**
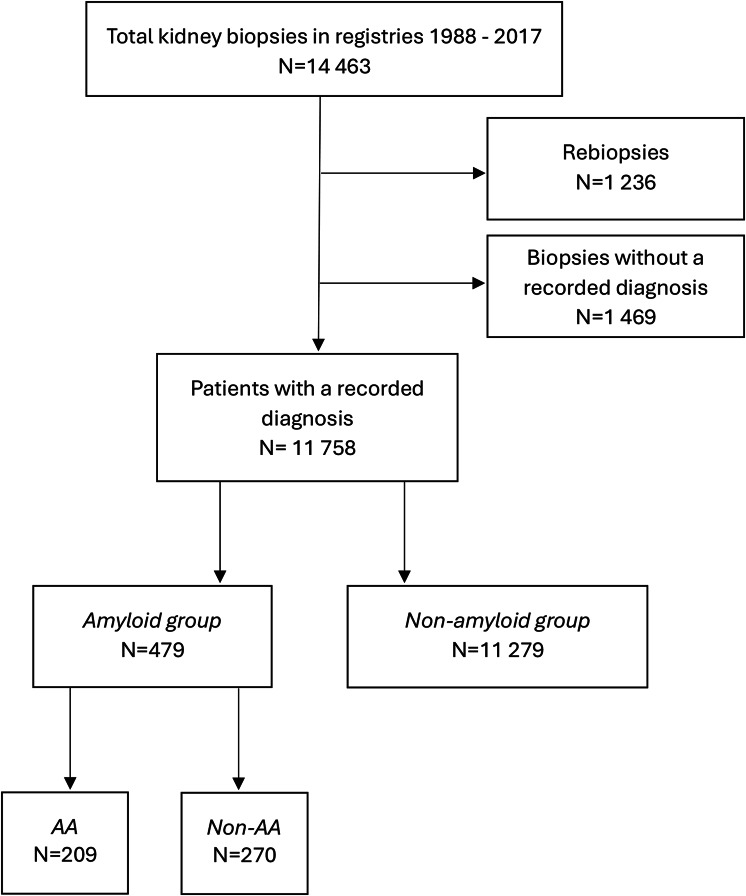
Study population. The national kidney registries, Norwegian Kidney Biopsy Register (NKBR) and The Norwegian Renal Registry (NRR), defined the overall study population. Patients registered with an amyloid containing kidney biopsy in NKBR or NRR from 1988 to 2017 constituted the kidney amyloidosis group. The remaining patients registered with a kidney biopsy with a diagnosis other than amyloid in these registries served as a comparator group. The amyloid group was divided into AA and non-AA for further comparison

Patients with amyloid in kidney biopsies registered in national registries from 1988 through 2017 were identified. The Norwegian Kidney Biopsy Register (NKBR) is a national registry containing clinical, biochemical, and histopathological data from native, non-neoplastic kidney biopsies from 1988 to 2015. From 2016 NKBR was merged into the Norwegian Renal Registry. Nephrologists report clinical and biochemical data to the registry, while an experienced nephropathologist records histopathology data. The registry coverage varies between 70 and 90% [27, 28]. The total number of kidney biopsies in the registries (*n* = 14 463) served as the basis for calculation of overall biopsy rate, while registry data from all other kidney biopsies except amyloidosis, served as comparator group (non-amyloid group). Incidence of amyloid in biopsies was calculated among eligible biopsies, excluding rebiopsies and biopsies without a recorded diagnosis (Fig. 1). Yearly population count reported as mid-year population and demographics for Norway were retrieved from Statistics Norway [29, 30].

We reviewed medical records of all included amyloidosis patients at the 26 hospitals reporting to the registries, thus confirming the diagnosis of amyloidosis, reducing missing data and obtaining data on amyloid type, additional laboratory analyses and underlying cause of AA amyloidosis. When typing data were available and conclusive, this determined the amyloid type, if not, clinician’s diagnosis was used to categorize the amyloid. If sufficient information was not available, the case was labelled ‘undetermined’. Data on amyloid typing in the study derive from pathology reports and review of medical records, and ambiguous cases have been discussed with the project’s nephropathologists. The evaluation of kidney biopsies in Norway is centralized to six hospitals. Congo red stain was used in all biopsies to detect amyloid depositions. In addition, the presence of amyloid was in most cases confirmed by electron microscopy, routinely performed on kidney biopsies in Norway. Amyloid in the study was mainly typed by immunostaining (IS): Immunohistochemistry (IHC) was used for AA, IHC or immunofluorescence (IF) for light chains (LC). IS for TTR have in the later years been used routinely in amyloid typing in four out of six nephropathology centres. Between 2010 and 2013, laser microdissection-mass spectrometry (LMD-MS) was used as a supplement at one of the hospitals in a few selected cases, but due to technical problems, the method was abandoned and only reintroduced in routine diagnostics in 2016/2017. For this study, the amyloid types were divided into amyloid A (AA) and non-amyloid A (non-AA) groups. The non-AA group comprised amyloid light-chain amyloidosis (AL), undetermined types, as well as other amyloid types.

### Definitions

*Proteinuria* in gram per 24 h (g/24 h) was calculated from available measurements as described by Vikse et al [31]. In 14 cases urine albumin-creatinine ratio (UACR) was the only available measurement and was converted to urine protein-creatinine ratio (UPCR) by multiplication by 2 if UACR < 70 and by 1.3 if UACR ≥ 70 [32]. *Absence of proteinuria* was defined as < 0.5 g/24 h [32, 33] and nephrotic range proteinuria as ≥ 3 g/24 h [11, 34]. *Nephrotic syndrome* was defined as proteinuria ≥ 3 g/24 h, albumin < 30 g/L and the presence of oedema [11, 34]. *Haematuria* was defined as blood 1 + or more on urine dipstick or haematuria reported as an indication for biopsy. *Serum creatinine* was converted to standardized creatinine according to isotope dilution mass spectrophotometry (IDMS)-traceable levels by reducing creatinine levels before 2005 with 5% [35, 36]. *Estimated glomerular filtration rate* (eGFR) was calculated using the CKD-EPI 2009 equation applying standardized creatinine and non-black race [37]. *Patients below 18 years* (*n* = 3) were excluded in the analysis of serum creatinine and their eGFR was determined by the bedside-Schwartz equation [38]. In one patient, aged 14 years, height was unavailable and was estimated using median expected height for age and gender [39]. *A cutoff of eGFR*< 60 and < 15 mL/min/1.73 m^2^, respectively, was used to define kidney insufficiency and end-stage kidney disease. eGFR ≥ 60 mL/min/1.73 m^2^ was labelled normal kidney function. *Hypertension* was defined as ≥140 mmHg systolic blood pressure (SBP) and/or ≥90 mmHg diastolic blood pressure (DBP) [40], and low blood pressure as SBP ≤ 100 mmHg. Corresponding to the European Society of Cardiology definition of hypertension in children, 16 years or older was used as a cut off for inclusion in the blood pressure analyses [40]. Older adults were defined as individuals above 65 years. For the purposes of comparison, the 30-year study period was divided into three *study decades* D1 (1988–1997), D2 (1998–2007) and D3 (2008–2017).

### Statistical analyses

The data were analysed using SPSS Statistics 29 (IBM Corp., Armonk, NY, United States) [41]. For analyses of non-amyloid versus amyloid group MedCalc Software Ltd Version 23.0.6 was used [42]. All tests were two-sided and *p-*values < 0.05 were considered significant. For continuous variables, Student’s t-test or one-way ANOVA were applied when parametric methods were suitable, while Independent-Samples Median test were used comparing not normally distributed variables. Categorical variables were compared using Pearson Chi-Square test or Fischer’s exact test where appropriate. Linear regression analysis was used to assess changes in biopsy rate per year. No multiple comparison adjustments were done as the purpose of the reported *p*-values is purely descriptive.

## Results

### Overall kidney biopsy rate and biopsy incidence of kidney amyloidosis

**Fig. 2:**
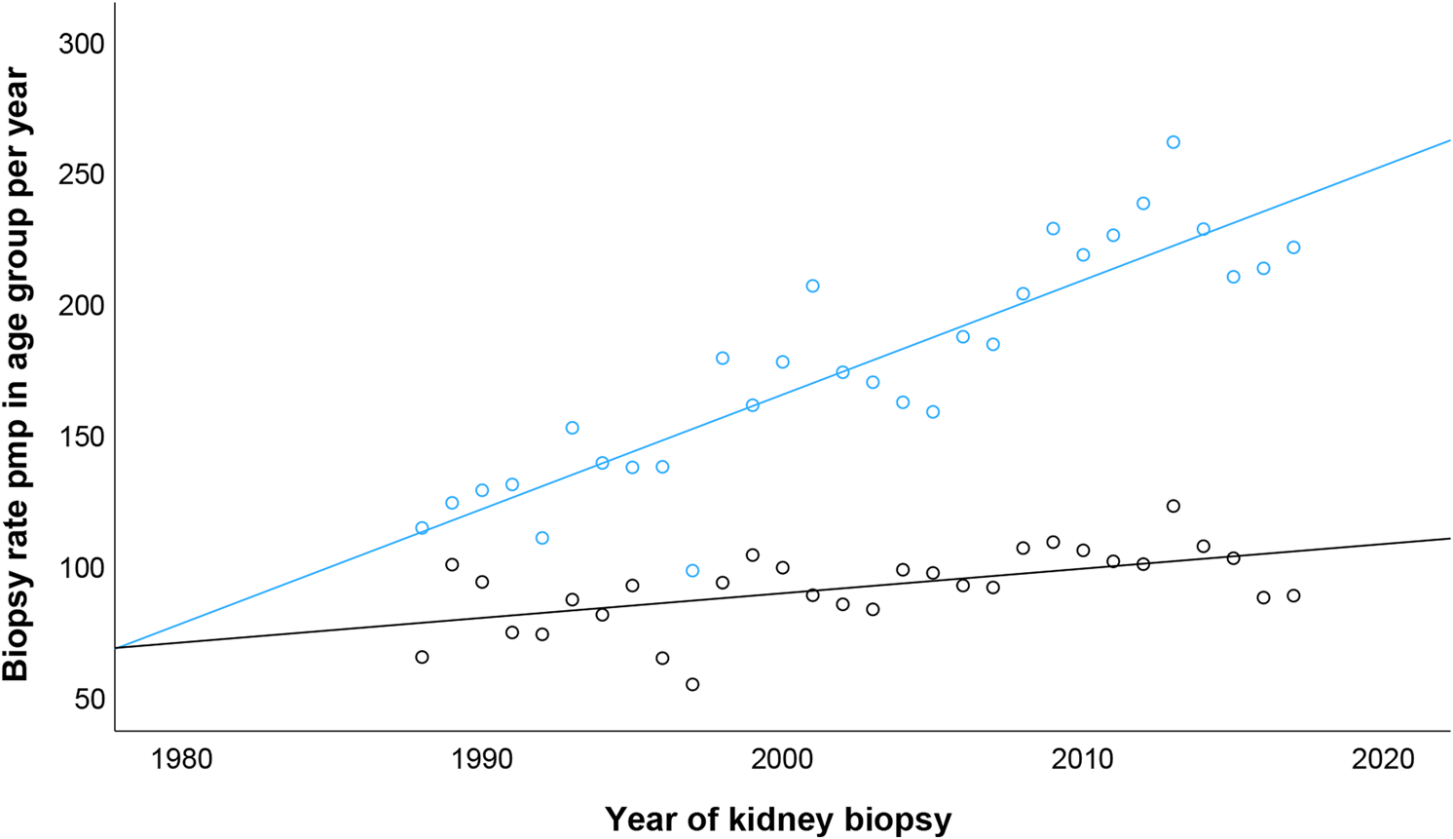
Kidney biopsy rate in the population respective of age group. The population was divided into two age groups: above 65 years (in blue) and 65 years and younger (in black). Kidney biopsy rate in the younger group increased with 0.94 biopsies per million population per year (pmp/y) (*p* = 0.01) while an increase of 4.36 biopsies pmp/y (*p* < 0.001) was seen in the older group

Overall, 14 463 kidney biopsies were registered in the 30-year study period between 1988 and 2017 (Fig. 1), giving an annual kidney biopsy rate between 60 and 142 kidney biopsies per million population per year (pmp/y) (Supplementary Figure S1). The 10-year average kidney biopsy rate increased from 85.3 to 120.0 biopsies pmp/y in the study period and the biopsy rate in older adults increased more than in the younger population (Fig. 2).

**Fig. 3:**
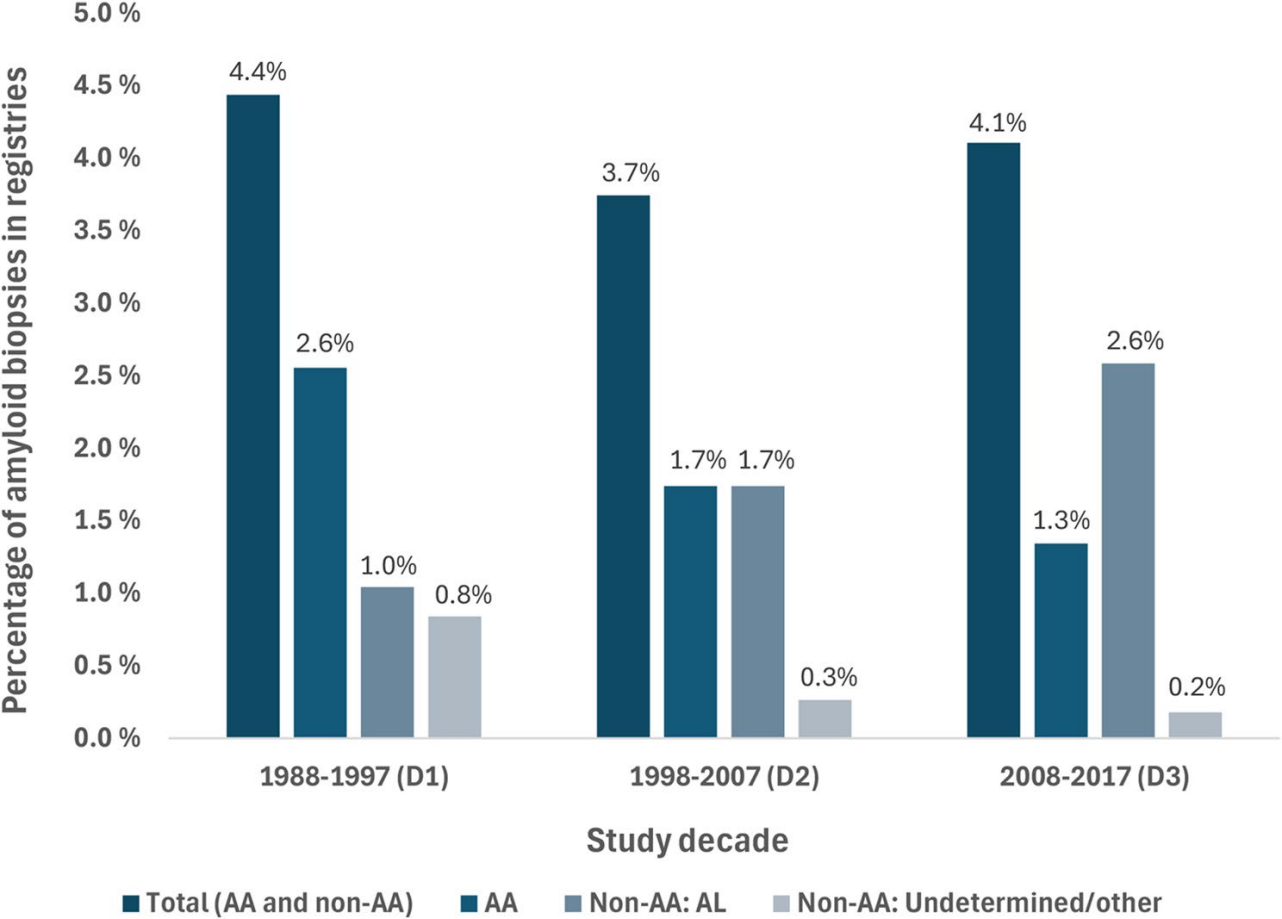
Biopsy incidence of kidney amyloidosis in the registries. The percentage of kidney amyloidosis in the registries’ kidney biopsies did not change significantly over the decades (*p* = 0.35). However, over time there was a significant change in the percentage of the underlying amyloid types of AA, AL and undetermined/other (*p* < 0.001). The composite group of non-AA (AL and undetermined/other) showed a similar significant increase from 1.9% in D1, 2.0% in D2 and 2.8% in D3 (*p* = 0.014)

The presence of amyloid was detected by Congo red stain. In six cases, there were only minute amounts of amyloid depositions that did not stain for Congo, but the diagnosis of amyloidosis was based on electron microscopy after finding the typical fibrillary structures with a diameter of 8–12 nm. Two of these six cases also had extra renal amyloid verified by Congo red staining in other organs (heart, bone marrow). A total of 479 kidney biopsies contained amyloid deposits, only one biopsy registered in NRR as kidney amyloidosis did not contain amyloid and was not included in the amyloid group. These 479 cases corresponded to 4.1% of all eligible kidney biopsies. In older adults, 7.4% of all eligible biopsies showed amyloidosis. Although the absolute number of cases increased over time, the proportion of amyloid biopsies in the registries did not change significantly (*p* = 0.35) (Fig. 3).

### Amyloid types

Non-AA amyloidosis was the largest group in our cohort with 270 (56.4%) cases and consisted of patients diagnosed with AL (83.7%), undetermined cases (14.1%) and other amyloid types (2.2%) (two cases of Fibrinogen A amyloidosis (AFib) [43], two cases of combined heavy chain–light chain amyloidosis (AH/AL), one case of LECT2 amyloidosis (ALECT2) and one case of Apolipoprotein A–IV amyloidosis (AApoA-IV)) (Table 1). Overall, 226 patients (47.2%) had an AL diagnosis, while 209 (43.6%) cases had AA. Of the AA cases, 138 (66.0%) were typed serum amyloid A (SAA) positive using immunohistochemistry (IHC) (Table 2). The remaining AA cases were classified by the treating physicians as AA based on the presence of AA-related diseases (Table 3). Serum and/or urine electrophoresis was performed in 63 of the 71 untyped AA cases, and no monoclonal protein (M-protein) was detected. In the typing of AL amyloidosis, immunostaining (IHC or IF) was used as the main method. Overall, typing attempts with either light chain immunostaining (LC–IS) and/or SAA immunohistochemistry (SAA-IHC) were observed in 88.1% of the 226 AL cases. Both LC–IS and SAA-IHC were used in typing of 73.5% of all AL cases, and in 93.8% (*n* = 166) of AL cases with attempted LC–IS typing, concurrent SAA-IHC was performed.

**Table 1:** Clinical data at diagnosis

	Whole study period 1988–2017 *N* = 479			Per decade D1-D3 D1: 1988–1997(N=132), D2: 1998–2007 (N=142), D3 2008–2017 (N=205)							
	AA	Non-AA	AA vs Non-AA	AA (*N* = 209)				Non-AA (*N* = 226)			
				D1	D2	D3	Change D1–D3	D1	D3	D3	Change D1–D3
**Amyloid type**											
**AA** n (%)	209 (43.6)			76 (57.6)	66 (46.5)	67 (32.7)	< 0.001				
**Non-AA**											
AL n (%))		226 (47.2)						31 (23.5)	66 (46.5)	129 (62.9)	< 0.001
Undetermined/other n (%)		44 (9.2)						25 (18.9)	10 (7.0)	9 (4.4)	< 0.001
**Age** at biopsy (years) median (IQR)	55.0 (24)	68.0 (13)	< 0.001	62.0 (27)	59.5 (25)	47.0 (11)	< 0.001^b^	68.5 (15)	70.0 (13)	68.0 (14)	0.566
**Older adults (> 65 years)** n (%)	64 (30.6)	157 (58.1)	< 0.001	31 (40.8)	25 (37.9)	8 (11.9)	< 0.001^b^	31 (55.4)	45 (59.2)	81 (58.7)	0.891
**Female** n (%)	93 (44.5)	141 (52.2)	0.093	42 (55.3)	33 (50.0)	18 (26.9)	0.002^b^	32 (57.1)	44 (57.9)	65 (47.1)	0.226
**Nephrotic syndrome** n (%)	106 (50.7)	189 (70.0)	< 0.001	34 (44.7)	29 (43.9)	43 (64.2)	0.028^b^	39 (69.6)	61 (80.3)	89 (64.5)	0.055
**No/minimal proteinuria** (< 0.5g/24h) n (%)	17 (8.1)	11 (4.1)	0.060	9 (11.8)	5 (7.6)	3 (4.5)	0.269	1 (1.8)	2 (2.6)	8 (5.8)	0.505
**Proteinuria** (g/24h) median (IQR)	5.1 (8.5)	6.0 (6.5)	0.242^a)^	3.6 (7.0)	3.5 (8.3)	8.6 (10.5)	< 0.001^b^	7.3 (9.2)	7.5 (6.5)	5.5 (6.3)	0.149
**Haematuria** n (%) †	110 (53.1)	165 (63.5)	0.024^b^	41 (53.9)	34 (52.3)	35 (53.0)	0.981	33 (62.3)	49 (68.1)	83 (61.5)	0.632
**eGFR** CKD-EPI 2009 (ml/min/1.73 m^2^) median (IQR)	27 (34)	53 (55)	< 0.001	32 (36)	31 (37)	21 (29)	0.043^b^	40 (67)	58 (57)	53 (49)	0.643
**Kidney insufficiency **(eGFR < 60 ml/min/1.73 m^2^) n (%)	172 (82.3)	153 (56.7)	< 0.001	61 (80.3)	52 (78.8)	59 (88.1)	0.317	36 (64.3)	42 (55.3)	75 (54.3)	0.430
**End-stage kidney disease** (eGFR < 15 ml/min/1.73 m^2^) n (%)	58 (27.8)	39 (14.4)	< 0.001	15 (19.7)	15 (22.7)	28 (41.8)	0.007^b^	15 (26.8)	12 (15.8)	12 (8.7)	0.005
**Urea** ≥ 30mmol/l n (%) †	22 (12.0)	15 (6.0)	0.025^b^	10 (17.5)	3 (5.0)	9 (13.6)	0.100^a)^	9 (19.1)	4 (5.7)	2 (1.5)	< 0.001
**Albumin** (g/l) mean (SD) †	26 (8)	25 (8)	0.203^a)^	27 (9)	27 (8)	24 (7)	0.067	24 (8)	23 (7)	27 (8)	0.002
**Haemoglobin** (g/dl) mean (SD) †	10.7 (2.1)	12.6 (2.1)	< 0.001	11.2 (2.0)	10.9 (2.0)	9.9 (2.1)	< 0.001^b^	12.0 (2.3)	12.4 (1.9)	12.9 (2.0)	0.020
**Erythrocyte sedimentation rate (ESR)** (mm/h) median (IQR) †	86 (50)	60 (49)	< 0.001	86 (44)	75 (48)	95 (43)	0.008^b^	80 (42)	62 (49)	53 (48)	0.029
**Systolic blood pressure (mmHg) ** mean (SD) *	137 (23)	129 (22)	< 0.001	142 (24)	138 (24)	132 (22)	0.025	131 (22)	129 (21)	128 (23)	0.637
**Diastolic blood pressure** (mmHg) mean (SD) †*	80 (13)	76 (12)	< 0.001	83 (13)	79 (14)	79 (14)	0.130^a)^	79 (12)	76 (12)	75 (12)	0.073
**Hypertension** (≥ 140/90) n (%) *	111 (53.4)	103 (38.1)	< 0.001	47 (62.7)	36 (54.5)	28 (41.8)	0.044	27 (48.2)	27 (35.3)	49 (35.5)	0.219
**Low blood pressure** (SBP ≤ 100) n (%) *	8 (3.8)	31 (11.5)	0.002	0 (0.0)	2 (3.0)	6 (9.0)	0.012	5 (8.9)	7 (9.2)	19 (13.8)	0.483

The table shows characteristics of the whole group (*n* = 479). The PWID group (*n* = 46) had markedly different characteristics from the rest of the AA cases, and sensitivity analyses were carried out to assess the extent of this group’s impact on overall group characteristics (Supplementary Table S1)

^a^*p*-value < 0.05 in sensitivity analyses excluding PWID

^b^*p*-value ≥ 0.05 in sensitivity analyses excluding PWID

^†^Missing: haematuria 12; urea 44; haemoglobin 6; albumin 3; ESR 48; diastolic blood pressure 1

*patients 16 years and older (*n* = 478)

**Table 2:** Amyloid typing

	Decade 1 (D1) 1988–1997	Decade 2 (D2) 1998–2007	Decade 3 (D3) 2008–2017	Change D1–D3 *p*-value	Whole period 1988–2017
**AA cases**	**76**	**66**	**67**		**209**
SAA-IHC performed and positive, *n* (%)	20 (26.3)	52 (78.8)	66 (98.5)	<0.001	138 (66.0)
LC–IS performed, *n* (%) (I)	1 (1.3)	13 (19.7)	47 (70.1)	<0.001	61 (29.2)
**AL cases**	**31**	**66**	**129**		**226**
Lambda IS positive, *n* (%) (II) and (III)	6 (19.4)	16 (24.2)	73 (56.6)	<0.001	95 (42.0)
Kappa IS positive, *n* (%) (IV)	1 (3.2)	9 (13.6)	24 (18.6)	0.080	34 (15.0)
LC–IS inconclusive or negative, *n* (%) (V) and (VI)	2 (6.5)	21 (31.8)	25 (19.4)	0.013	48 (21.2)
LC–IS not performed, SAA-IHC negative *n* (%) (VII)	3 (9.7)	15 (22.7)	4 (3.1)	<0.001	22 (9.7)
No IS performed, *n* (% of AL in decade) (VIII)	19 (61.3)	5 (7.6)	3 (2.3)	<0.001	27 (11.9)
**Undetermined (Utd) cases**	**25**	**10**	**3**		**38**
Typing attempt with SAA-IHC and LC–IS, *n* (%) (IX)	2 (8.0)	5 (50.0)	2 (66.7)	0.004	9 (23.7)
Typing attempt with SAA-IHC or LC–IS only *n* (%) (X)	2 (8.0)	4 (40.0)	0 (0.0)	0.065	6 (15.8)
No typing attempt, *n* (%) (XI)	21 (84.0)	1 (10.0)	1 (33.3)	<0.001	23 (60.5)
**Other**	**0**	**0**	**6**		**6**
Typed using IHC and LMD-MS, *n* (%) (XII)	0 (0.0)	0 (0.0)	6 (100.0)		6 (100.0)

*AA-group* All 138 AA cases with SAA-IHC performed were SAA positive, in 61 LC–IS was also performed. All 71 untyped AA cases had AA-related disease, 63 of these had serum and/or urine electrophoresis performed with no M-protein detected. *Non-AA group* AL cases: A positive immunostaining for kappa or lambda was found in 129 biopsies. If not explicitly stated, SAA-IHC was performed in all AL biopsies except eleven cases noted in Supplementary Table S2(III–V). The remaining cases were classified as AL based on clinical presentation and findings, including factors as M-protein, negative SAA and/or absence of AA-related diseases into the assessment. *Non-AA group: Undetermined cases* Available typing and clinical information did not provide enough information to classify the amyloid. *Further diagnostic assessments* of the patient material are described in Supplementary Table S2 (I, II, VI–XII). *Abbreviations* SAA = serum amyloid A, IHC = immunohistochemistry, IF = immunofluorescence, LC = light chain, IS = immunostaining (IHC or IF)

**Table 3:** Causes of AA amyloidosis

	Decade 1 (D1) 1988–1997	Decade 2 (D2) 1998–2007	Decade 3 (D3) 2008–2017	Change D1–D3 *p*-value	Whole period 1988–2017
**Rheumatic disease *n ***(%)	64 (84.2)	47 (71.2)	12 (17.9)	<0.001	123 (58.9)
**Inflammatory bowel disease (IBD) *n*** (%)	3 (3.9)	7 (10.6)	4 (6.0)	0.273	14 (6.7)
**Chronic infection *n*** (%)	7 (9.2)	4 (6.1)	3 (4.5)	0.507	14 (6.7)
**Injecting drug use *n*** (%)	0 (0.0)	3 (4.5)	43 (64.2)	<0.001	46 (22.0)
**Other *n*** (%)	1 (1.3)	0 (0.0)	3 (4.5)	0.209	4 (1.9)
**Unknown *n*** (%)	1 (1.3)	5 (7.6)	2 (3.0)	0.156	8 (3.8)

Inflammatory bowel disease (IBD) was found in a total of 20 patients (9.6%), but due to overlapping disorders, it was the sole cause in only 14 (6.7%). Likewise, chronic infection (not related to drug use) was a possible cause in a total of 27 patients (12.9%), but in only 14 patients (6.7%) was chronic infection a stand-alone cause

### Changes in typing practices and amyloid types over time

The scope and the precision of the amyloid typing improved significantly from D1 to D3 (Table 2). A significant increase in AA typed positive for SAA-IHC was seen throughout the decades (*p* < 0.001). Notably, 78.9 % (*n* = 56) of the untyped AA cases were in the first study decade (D1), and in D3 98.4% of all AA were typed using SAA-IHC. Among the 226 cases classified as AL, cases without any typing attempts (*n* = 27) decreased significantly from 61.3% in D1 to 2.3% in D3 (*p* < 0.001). Parallel to this, there was a significant increase in cases typed as AL lambda from 19.4% in D1 to 56.6% in D3 (*p* < 0.001), and a non-significant increase in typed AL kappa (3.2% in D1 to 18.6% in D3, *p* = 0.080). Moreover, AL cases typed with both LC–IS and SAA-IHC increased significantly from 22.6% in D1 to 89.1% in D3 (*p* < 0.001). In the undermined group, there was a similar tendency towards more typing, as 91.3% of cases without any typing attempts occurred in D1.

Over the three decades we observed a relative increase in non-AA (*p* < 0.001) (Table 1). From D1 to D3, non-AA increased from 1.9% to 2.8% of all eligible kidney biopsies (*p* = 0.014), while AA decreased from 2.6% to 1.3% (*p* < 0.001) (Fig. 3). While rheumatic disease was the most prevalent cause of AA overall and present in 58.9% of all AA cases, this subgroup decreased from 84.2% of AA cases in D1 to 17.9% in D3 (*p* < 0.001) (Table 3). Conversely, amyloidosis in people who inject drugs (PWID), emerged in D2, and became the most important cause of AA (64.2%) in D3 (*p* < 0.001). Other causes of AA did not change significantly over time (Table 3).

### Clinical features at diagnosis

Proteinuria (≥0.5 g/24 h) was the most common feature and found in 94.2% of all patients with amyloidosis at diagnosis (Table 1). Nephrotic range proteinuria was present in 76.0% and nephrotic syndrome in 61.6% whereas only in 17.6% of the non-amyloid comparator group (*p* < 0.001). Notably, 13.5% of all registered kidney biopsied patients with nephrotic syndrome had amyloidosis, while in older adults with nephrotic syndrome, amyloidosis accounted for 25.2% of cases. None of the amyloidosis patients had nephritic syndrome, although haematuria was common (58.9%). Kidney function was normal in 32.2% of amyloid cases and more common in the non-AA group (43.3% vs 17.7%, *p* < 0.001).

Both mean systolic blood pressure (SBP) and diastolic blood pressure (DBP) in the amyloid group were in the normal range and significantly lower than in the non-amyloid group (SBP mean ± SD 132 ± 23 vs 142 ± 24 mmHg, *p* < 0.001 and DBP 78 ± 13 vs 83 ± 14 mmHg, *p* < 0.001). Likewise, the prevalence of hypertension in the amyloid group of 44.8% contrasted the 57.4% in the non-amyloid group (*p* < 0.001). In both groups, average SBP and DBP at diagnosis decreased over the decades (*p* < 0.001). The percentage of individuals with low blood pressure (≤100 mmHg) increased from 3.8% to 12.2% (*p* = 0.015) and low blood pressure was present in 8.2% of all amyloid cases while only in 2.0% of the non-amyloid group (*p* < 0.001). Overall, 16.7% of all patients ≥ 16 years with low blood pressure at diagnosis had amyloidosis. Notably, of the 39 amyloid cases with low blood pressure, 79.5% were in the non-AA group.

### Clinical presentation of the amyloid types

Non-AA and AA differed in their clinical presentation (Table 1). AA patients were younger (*p* < 0.001), had lower prevalence of nephrotic syndrome (*p* < 0.001), lower kidney function (*p* < 0.001) and more hypertension (*p* < 0.001). Conversely, the non-AA group more often had low blood pressure (*p* = 0.002), higher haemoglobin levels (*p* < 0.001) and lower erythrocyte sedimentation ratio (ESR) (*p* < 0.001).

### Amyloidosis in people who inject drugs (PWID)

The AA group included a subgroup of PWID (*n* = 46). This group had distinct demographic and clinical features distinguishing them from the remaining AA group. Group characteristics of the amyloidosis group excluding PWID are shown in Supplementary Table S1. The PWID-AA group were younger at diagnosis compared to the other AA patients (median age (IQR) 46.0 (9) years vs 61.0 (25) years, *p* < 0.001) and predominantly male (73.9% vs 50.3%, *p* < 0.004). Moreover, compared to other AA patients they often presented with nephrotic syndrome (71.7% vs 44.8%, *p* = 0.001), lower kidney function (median eGFR (IQR) 16 (21) vs 32 (39) mL/min/1.73 m^2^, *p* = 0.005) and more often end-stage kidney disease (45.7% vs 22.7%, *p* < 0.002).

## Discussion

In our 30-year cohort, we identified 479 cases of biopsy-proven kidney amyloidosis. While overall kidney biopsy rate increased, the percentage of registry biopsies with kidney amyloidosis per decade remained stable. However, there was a shift in types, with an increase in non-AA and a decrease in AA cases, although the emergence of AA amyloidosis in PWID attenuated the AA decline. Kidney amyloidosis patients almost invariably had proteinuria; and kidney insufficiency and nephrotic syndrome were common. Compared to other kidney biopsy patients, hypertension was less prevalent and low blood pressure more common. Comparing AA and non-AA patients, there were clear clinical differences which agree with previous reports [11, 13, 14]. Patients in the AL-dominated non-AA group were older and more likely to have nephrotic syndrome, while the AA group had more impaired kidney function and higher blood pressure at diagnosis.

There is a paucity of larger population-based studies focusing on kidney amyloidosis. Several studies from large referral centres examine systemic amyloidosis in general [19, 20, 44], however, to our knowledge only three studies focus on kidney amyloidosis in a population-based context [12–14]. The largest study is from 2013 and includes 653 cases from the Spanish Registry of Glomerulonephritis [13], while the most recent, from 2017, reports on 281 cases from the Japan Renal Biopsy Registry [12]. Both studies rely on registry data alone and the latter cannot provide data on amyloid type. Bergesio et al reported on 373 cases from selected Italian nephrological units, also including patients diagnosed with non-kidney biopsies [14]. Our study is a nationwide cohort (Norway) with strict inclusion criteria (kidney biopsy only) and contains supplementary amyloid relevant data from medical records.

The biopsy incidence of kidney amyloidosis in our study was 4.1%, which agrees with 1.3–4.5% reported in previous reports [12–14, 45–47]. The predominant group was non-AA (56%) where AL constituted 84%. This is in accordance with the well-known predominance of AL in developed countries [2, 3, 20]. In addition, after reviewing patient history, laboratory work and inconclusive typing attempts of the 38 undetermined cases, we estimate 60% of these to be probable AL cases where data were insufficient for a definite diagnosis.

A shift in amyloid types occurred despite stable overall biopsy incidence, with an increase in non-AA and a decrease in AA. Although larger epidemiological studies have not found significant changes in the incidence of systemic AL amyloidosis [48–50], increasing incidence of AL amyloidosis affecting the kidney has previously been shown in studies from Taiwan and Brazil [25, 51]. Similarly, increasing incidence of non-type-specific kidney amyloidosis was found in 1996–2000 in Italy [14]. Our observed rise in non-AA cases may be influenced by several factors. Amyloidosis is believed to be underdiagnosed [1], and increased biopsy rate may permit diagnosis of more cases. As amyloidosis is more prevalent in older individuals, the raised biopsy rate among older adults may contribute [12, 50]. Finally, several authors hypothesize an impact of increased amyloid awareness [14, 19, 25, 50]. In alignment with this, our findings suggest patients in recent years may be diagnosed at an earlier stage. For non-AA, significant improvements were seen in serum albumin, haemoglobin and ESR at diagnosis. Additionally, the percentage of patients with non-AA presenting with end-stage kidney disease fell from 26.8% to 8.7% (*p* = 0.005) and the proportion of patients presenting with high urea (≥30 mmol/l) fell significantly over time in the overall, non-AA, and AA-excluding-PWID groups. Overall, this might reflect earlier diagnosis and better amyloid awareness.

The decline in AA amyloidosis in developed countries in recent years has been described broadly in the literature [13, 19–21]. A similar trend is indicated here, with reduction in AA caused by rheumatic disease. However, the recent rise in AA cases in PWID has slowed the overall AA decline. A study from National Amyloidosis Centre (NAC) in the United Kingdom identified the same pattern [19]. The occurrence of AA in PWID is a growing concern. Connolly et al observed an increase in PWID with AA in London between 1990 and 2005 [52]. Similarly, in a study from San Francisco from 1998 to 2013 the overall incidence of amyloidosis on kidney biopsy was 6.6% (28 patients), where 86% had a history of intravenous drug use and AA on biopsy [53]. The clinical presentation of PWID in our study, differed from other AA patients with higher incidence of nephrotic syndrome, lower kidney function and more frequent end-stage kidney disease. In line with this, we found an increase in end-stage kidney disease in D3 (Table 1) which was no longer statistically significant when excluding PWID (Supplementary Table S1). In their study of the changing epidemiology of AA amyloidosis, Lane et al also found an unexpected rise in end-stage kidney disease from first to third cohort alongside an increase in PWID from 1% to 13% [19]. The advanced presentation of AA in PWID likely has multifactorial causes. Delayed healthcare seeking, poor medication adherence, and, thus, limited access to kidney-protective treatment, may all play a role. Another factor could be drug-related kidney injury [54–56], and several biopsies in our cohort showed acute tubulointerstitial nephritis and some had acute tubular necrosis. Heroin-induced focal segmental glomerulosclerosis cannot be entirely excluded, but no histopathological signs of vasculitis, thrombotic microangiopathy or immune complex-related diseases were noted. Overall, amyloidosis remains an important cause of kidney disease in this patient population [57], and heightened awareness of this condition in PWID is important.

Proteinuria was observed in almost all amyloidosis patients, and nephrotic syndrome was common. This is consistent with previous reports [11–15, 25, 47]. Moreover, in patients with nephrotic syndrome, amyloidosis was a prevalent finding, particularly among older adults. Thus, the presence of nephrotic syndrome in any patient, and especially in older adults, should prompt the clinician to consider amyloidosis. Only 5.8% of the patients in our study presented with no or minimal proteinuria (<0.5 g/24 h). This unusual phenotype without overt proteinuria has been previously described in both AA and AL patients with a vascular-limited amyloid deposition pattern [58, 59]. Lack of awareness of the vascular-limited phenotype and proteinuria being a significant factor in the decision to perform a renal biopsy, might contribute to underdiagnosis of this subgroup [59]. Kidney insufficiency was common, as also previously reported [11, 12, 14, 45]. Our finding of a high prevalence of haematuria is in line with recent reports from Japan and Spain [12, 13] and contrasts previous beliefs that haematuria is uncommon in kidney amyloidosis [60]. This emphasises the importance of recognizing haematuria as a common finding in this condition, and that its presence does not exclude amyloidosis. Hypertension is a prevalent feature of kidney disease, but less so of amyloidosis [12, 15, 25]. Low blood pressure and orthostatic hypotension are features of AL, attributed to factors such as cardiac involvement or autonomic dysfunction [2, 17]. Supporting this, we found significantly less hypertension and more often low blood pressure in the amyloid group than the non-amyloid group. Moreover, as expected, low blood pressure was more prevalent in the non-AA group. Over time, prevalence of hypertension at diagnosis decreased in both amyloid and non-amyloid groups. This trend, also observed in the Norwegian population-based HUNT study, may result from overall improved blood pressure in the population [61].

Non-AA amyloidosis can be challenging to type due to false negative light chains staining and absence of routine testing for rare amyloid types. Immunostaining with both SAA-IHC and LC–IS, regardless of clinical suspicion, reduces the misclassification risk [62]. This dual typing (LC and SAA) significantly increased from D1 to D3 (*p* < 0.001) (Table 2). However, some false positives remain possible. In the undetermined group, limited typing increases misclassification risk, though access to clinical information helps mitigate this. SAA-IHC on formalin-fixed tissue has in our experience high sensitivity and specificity for AA amyloidosis, with a minimal risk of false positive AA [63, 64]. Among 346 cases stained for AA, there were only one false positive (typed AL lambda with LMD-MS) and three inconclusive staining (Supplementary Table S2). While immunostaining has its pitfalls, LMD-MS has become a gold standard in recent years, though its availability remains limited [65, 66]. The reduction of undetermined cases and identification of six ‘other’ amyloid cases in D3 (Table 2), highlight the progress in amyloid typing over the 30 years and demonstrate how concurrent SAA-IHC and LC–IS, supplemented with LMD-MS, improve amyloid diagnostics.

There are several limitations to our study. Given the 30-year span of this cohort, retyping of all biopsies using the current typing gold standard of LMD-MS is neither feasible nor would it reflect the diagnostic standards at time of biopsy. While we cannot rule out some misclassification, comprehensive typing data and clinical context minimize this risk. Moreover, amyloid detection with Congo red stain may in certain circumstances yield false positive results, however, in the hands of experienced pathologists and with the use of supplementary electron microscopy, the proportion of false positives is very low [67]. Thus, the centralization of nephropathology in Norway to six centres, helps to improve the accuracy of both amyloid detection and typing in kidney biopsies. Another limitation is related to our study population. The population is defined by the registry reported cases and is thus reliant on clinicians’ reports to the registry. Despite the high registry coverage [27, 28], not all cases of kidney biopsy-proven kidney amyloidosis are captured. Furthermore, the study does not include kidney amyloidosis diagnosed from non-kidney biopsies where kidney involvement is inferred from proteinuria or kidney insufficiency [33]. Additionally, the acquisition of supplementary data from medical records was done retrospectively, and inevitably, in some cases data were no longer available. However, the study design using a combination of registry data reported prospectively by clinicians and retrospective review of medical records gaining additional amyloid relevant information, is a study strength. Moreover, the large study size and the study’s nationwide population basis are other strengths, which are purposeful to shed light on a rare disease such as kidney amyloidosis.

In conclusion, kidney amyloidosis shows a diverse clinical presentation, but proteinuria is a common denominator. Nephrotic syndrome is frequently observed, and among older adults, amyloidosis is a common cause of nephrotic syndrome. Early diagnosis is important, and clinicians should pay particular attention to patients at risk and maintain a high degree of amyloid awareness, especially in patients with kidney disease and unexplained hypo- or normotension, proteinuria or nephrotic syndrome. Precise amyloid typing is a cornerstone, and access to robust typing methods is important. Lastly, the observed shifts in amyloid types are noteworthy trends that may have implications for future identification of patients as well as upcoming healthcare demands.

## Electronic supplementary material

Below is the link to the electronic supplementary material.

Supplementary Material 1

Supplementary Material 2

Supplementary Material 3

## Data Availability

De-identified data may be shared upon reasonable request and after application to the Regional Committee for Medical and Health Research Ethics (REK Sørøst, Norway), in cooperation with the authors. Requests to access the dataset should be directed to Tale Norbye Wien (twien@vestreviken.no).
