# Supplementary material for: Epidemiology and clinical presentation of kidney amyloidosis have changed over the past three decades: a nationwide population-based study
Source: BMC Nephrol. 2025 Jun 2;26:272. doi: 10.1186/s12882-025-04136-w (PMC12131639; doi:10.1186/s12882-025-04136-w)
Supplement: Supplementary file 1 — Supplementary Material 1 [file 12882_2025_4136_MOESM1_ESM.pdf]

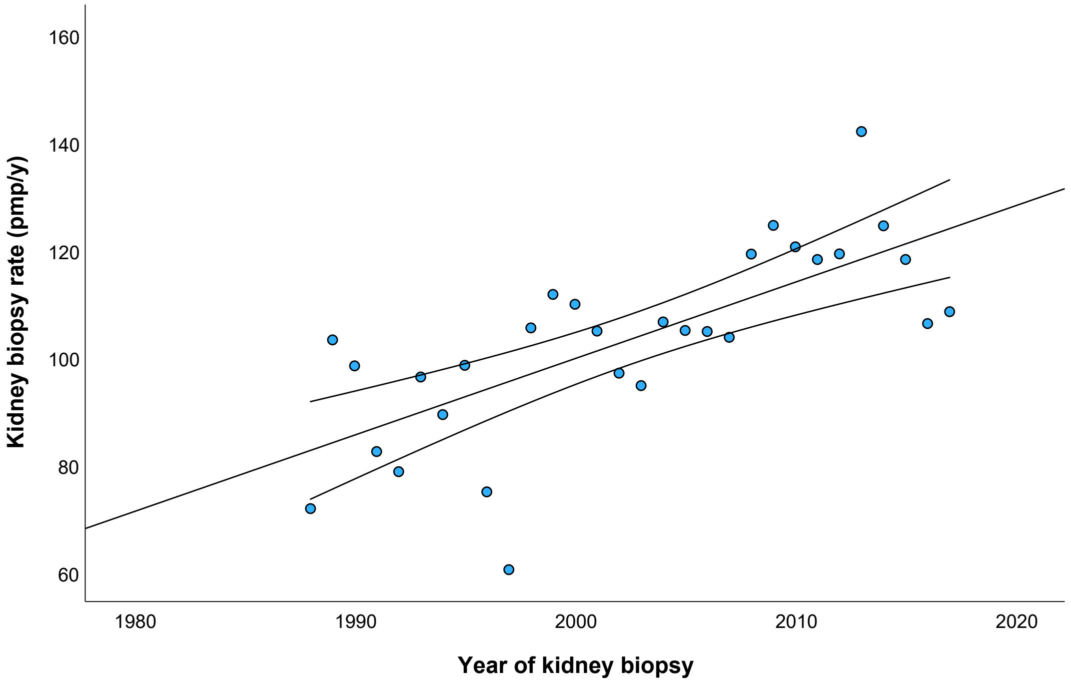

Supplementary Figure S1: Kidney biopsy rate in overall population (Norway). Kidney biopsy rate in the overall population increased by 1.42 biopsies per million population per year (pmp/y) in the period from 1988 to 2017 (p<0.001)
