# Supplementary material for: Epidemiology and clinical presentation of kidney amyloidosis have changed over the past three decades: a nationwide population-based study
Source: BMC Nephrol. 2025 Jun 2;26:272. doi: 10.1186/s12882-025-04136-w (PMC12131639; doi:10.1186/s12882-025-04136-w)
Supplement: Supplementary file 2 — Supplementary Material 2 [file 12882_2025_4136_MOESM2_ESM.docx]

|  | **Whole study period**  **1988 – 2017**  N=433 | | | **Per decade D1-D3**  **D1: 1988 – 1997, D2: 1998 – 2007, D3 2008 – 2017**  N=132 N= 139 N=162 | | | | | | | |
| --- | --- | --- | --- | --- | --- | --- | --- | --- | --- | --- | --- |
|  | **AA** | **Non-AA** | **AA vs Non- AA** | **AA** (N=163) | | | | **Non-AA** (N=226) | | | |
|  |  |  |  | **D1** | **D2** | **D3** | **Change D1-D3** | **D1** | **D3** | **D3** | **Change**  **D1-D3** |
| **Amyloid type**  **AA** n (%)    **Non-AA**  AL n (%)  Undetermined/other n (%) | 163 (37.6) | 226 (52.2)  44  (10.2) |  | 76  (57.6) | 63  (45.3) | 24 (14.8) | <0.001 | 31 (23.5)  25 (18.9) | 66  (47.5)  10  (7.2) | 129 (79.6)  9  (5.6) | <0.001  <0.001 |
| **Age** at biopsy (years)  median (IQR) | 61.0 (25) | 68.0 (13) | <0.001 | 62.0 (27) | 60.0 (22) | 57.0  (49) | 0.477 | 68.5 (15) | 70.0 (13) | 68.0  (14) | 0.566 |
| **Older adults (> 65 years)**  n (%) | 64  (39.3) | 157 (58.1) | <0.001 | 31 (40.8) | 25 (39.7) | 8 (33.3) | 0.805 | 31 (55.4) | 45 (59.2) | 81 (58.7) | 0.891 |
| **Female**  n (%) | 81 (49.7) | 141 (52.2) | 0.610 | 42 (55.3) | 32 (50.8) | 7 (29.2) | 0.081 | 32 (57.1) | 44 (57.9) | 65 (47.1) | 0.226 |
| **Nephrotic syndrome**  n (%) | 73 (44.8) | 189 (70.0) | <0.001 | 34 (44.7) | 26 (41.3) | 13 (54.2) | 0.557 | 39 (69.6) | 61 (80.3) | 89 (64.5) | 0.055 |
| **No/minimal proteinuria**  (<0.5g/24h) n (%) | 17 (10.4) | 11 (4.1) | 0.009 | 9  (11.8) | 5  (7.9) | 3 (12.5) | 0.730 | 1  (1.8) | 2  (2.6) | 8  (5.8) | 0.505 |
| **Proteinuria** (g/24h)  median (IQR) | 3.6 (6.6) | 6.0  (6.5) | <0.001 | 3.6  (7.0) | 3.3  (6.2) | 4.9 (6.2) | 0.652 | 7.3  (9.2) | 7.5  (6.5) | 5.5  (6.3) | 0.149 |
| **Haematuria**  n (%) † | 94 (58.0) | 165 (63.5) | 0.265 | 41 (53.9) | 34 (54.8) | 19 (79.2) | 0.075 | 33 (62.3) | 49 (68.1) | 83 (61.5) | 0.632 |
| **eGFR** CKD-EPI 2009 (ml/min/1.73 m^2^) median (IQR) | 32 (39) | 53  (55) | <0.001 | 32  (36) | 31  (36) | 37 (45) | 0.737 | 40  (67) | 58  (57) | 53  (49) | 0.643 |
| **Kidney insufficiency** (eGFR < 60 ml/min/1.73 m^2^) n (%) | 130 (79.8) | 153 (56.7) | <0.001 | 61 (80.3) | 51 (81.0) | 18 (75.0) | 0.804 | 36 (64.3) | 42 (55.3) | 75 (54.3) | 0.430 |
| **End-stage kidney disease** (eGFR < 15 ml/min/1.73 m^2^) n (%) | 37 (22.7) | 39 (14.4) | 0.029 | 15 (19.7) | 15 (23.8) | 7 (29.2) | 0.608 | 15 (26.8) | 12 (15.8) | 12 (8.7) | 0.005 |
| **Urea** ≥30mmol/l  n (%) † | 13 (9.5) | 15  (6.0) | 0.197 | 10 (17.5) | 3  (5.3) | 0  (0.0) | 0.028 | 9  (19.1) | 4  (5.7) | 2  (1.5) | <0.001 |
| **Albumin** (g/l)  mean (SD) † | 27  (7) | 25  (8) | 0.034 | 27  (9) | 28  (8) | 26  (9) | 0.704 | 24  (8) | 23  (7) | 27  (8) | 0.002 |
| **Haemoglobin** (g/dl)  mean (SD) † | 11.1 (2.0) | 12.6 (2.1) | <0.001 | 11.2 (2.0) | 11.0 (2.0) | 10.7 (2.1) | 0.570 | 12.0 (2.3) | 12.4 (1.9) | 12.9 (2.0) | 0.020 |
| **Erythrocyte sedimentation rate (ESR)** (mm/h) median (IQR) † | 77 (50) | 60  (49) | <0.001 | 86  (44) | 74  (48) | 77 (57) | 0.398 | 80  (42) | 62  (49) | 53  (48) | 0.029 |
| **Systolic blood pressure** (mmHg)  mean (SD) * | 139 (24) | 129  (22) | <0.001 | 142  (24) | 139  (23) | 127 (22) | 0.027 | 131  (22) | 129  (21) | 128  (23) | 0.637 |
| **Diastolic blood pressure** (mmHg)  mean (SD) †* | 80 (13) | 76  (12) | <0.001 | 83  (13) | 80  (14) | 75 (12) | 0.014 | 79  (12) | 76  (12) | 75  (12) | 0.073 |
| **Hypertension** (≥140/90 mmHg)  n (%) * | 90 (55.6) | 103 (38.1) | <0.001 | 47 (62.7) | 35 (55.6) | 8 (33.3) | 0.042 | 27 (48.2) | 27 (35.3) | 49 (35.5) | 0.219 |
| **Low blood pressure** (SBP ≤ 100 mmHg) n (%) * | 5  (3.1) | 31 (11.5) | 0.002 | 0  (0.0) | 1  (1.6) | 4 (16.7) | <0.001 | 5  (8.9) | 7  (9.2) | 19 (13.8) | 0.483 |

**Supplementary Table S1 Clinical data at diagnosis excluding AA in people who inject drugs (PWID)**

†) Missing: haematuria 11; urea 44; haemoglobin 6; albumin 3; ESR 44; diastolic blood pressure 1; *) patients 16 years and older (n=432)
