# Supplementary material for: Epidemiology and clinical presentation of kidney amyloidosis have changed over the past three decades: a nationwide population-based study
Source: BMC Nephrol. 2025 Jun 2;26:272. doi: 10.1186/s12882-025-04136-w (PMC12131639; doi:10.1186/s12882-025-04136-w)
Supplement: Supplementary file 3 — Supplementary Material 3 [file 12882_2025_4136_MOESM3_ESM.docx]

**Supplementary Table S2** **Detailed information on cases with ambiguous and/or incomplete amyloid typing**

| *AA group* | *I: LC-IS was performed in 61 patients with AA and SAA-IHC positive:*  LC-IS was negative in 57 biopsies, and ambiguous in four biopsies. All four cases with ambiguous LC-IS had AA-related conditions (two PWID, two rheumatic disease) and all were categorized as AA by their clinician. One had also monoclonal kappa light chains in serum. |
| --- | --- |
| *Non-AA group: AL* | *II: Among four biopsies positive for lambda-IS:*  One biopsy was positive for both SAA-IHC and lambda IS, LMD-MS confirmed AL lambda. Three biopsies were assessed likely lambda positive, but not entirely conclusive staining. All three were negative for SAA-IHC and had lambda isotype M-protein. In two, LMD-MS was done and confirmed AL lambda. |
|  | *III: Among four biopsies positive for lambda-IS without SAA-IHC performed:*  All had M-protein of lambda isotype. Three had no AA-related diseases, one had mild ankylosing spondylitis and lambda light chain multiple myeloma. |
|  | *IV: One kappa positive biopsy without SAA-IHC:*  The patient had no AA-related disease, M-protein (kappa isotype) was present, and the patient was diagnosed with kappa multiple myeloma. |
|  | *V: Among six biopsies inconclusive/negative for LC-IS without SAA-IHC performed; classified as AL:*  All had M-protein (including two with multiple myeloma), one of the six patients had AA-related disease. |
|  | *VI: Among 42 inconclusive/negative LC-IS biopsies also typed with SAA-IHC; classified as AL:*  SAA-IHC was performed in all 42, and negative in 41. One biopsy had inconclusive, weak SAA-staining. This patient had M-protein of lambda isotype and clinician’s diagnosis was AL. |
|  | *VII: Among 22 biopsies not typed with LC-IS, and negative for SAA-IHC; classified as AL*:  All were treated as AL by the clinician. M-protein was present in 20 patients. |
|  | *VIII: Among 27 biopsies classified as AL no typing was performed:*  AL was diagnosed on clinical basis and all had M-protein. Two patients had mild possible AA-related diseases (unspecified rheumatism and chronic airway infections). |
| *Non-AA group: Undetermined* | *IX: Among nine biopsies typed with both SAA-IHC and LC-IS; amyloid classified as undetermined*:  One biopsy stained weakly for SAA with negative LC-IS. The patient had no AA related disease, and no information on M-protein was available. The remaining eight biopsies had negative SAA-IHC and negative/inconclusive LC-IS. Three patients had M-protein; in five none was detected (no FLC assay performed). Two patients had possible AA-related disease (but negative SAA-IHC): in one case LMD-MS was performed with inconclusive result, but no indication of AA. |
|  | *X: Among six biopsies attempted typed with SAA-IHC or LC-IS; amyloid classified as undetermined*:  One biopsy was typed with LC-IS only (negative), had no M-protein, a possible AA-related disease (infection), but no definite AA-diagnosis. Four biopsies were typed with SAA-IHC only (negative): M-protein was detected in two, absent in two (no FLC assay). One biopsy had inconclusive SAA-IHC, no M-protein and no AA-related disease. |
|  | *XI: Among twenty-three biopsies not typed;* *amyloid classified as undetermined*:  Five had M-protein, five had possible AA-related disease, and of these, two had both conditions. Six patients had not sufficient tests to exclude M-protein. In five, tests were not available/not performed. |
| *Non-AA group: Other* | *XII: Six biopsies; amyloid classified as other:*  Two AFib, two AH/AL, one ALECT2 and one AApoA-IV |

Supplementary Table S2 shows typing data in relation to clinical data where relevant. Please refer to Table 3 for a general overview of typing data. IS= immunostaining (IHC or IF), LC= light chain, SAA =serum amyloid A, FLC= free light chain, AFib = Fibrinogen amyloidosis, AH/AL = combined heavy chain – light chain amyloidosis, ALECT2 = LECT2 amyloidosis, AApoA-IV = ApoA-IV amyloidosis.
